# Discovery of Novel *Backusella* (Backusellaceae, Mucorales) Isolated from Invertebrates and Toads in Cheongyang, Korea

**DOI:** 10.3390/jof7070513

**Published:** 2021-06-27

**Authors:** Thuong T.T. Nguyen, Kerstin Voigt, André Luiz Cabral Monteiro de Azevedo Santiago, Paul M. Kirk, Hyang Burm Lee

**Affiliations:** 1 Environmental Microbiology Laboratory, Department of Agricultural Biological Chemistry, College of Agriculture & Life Sciences, Chonnam National University, Gwangju 61186, Korea; ngthuongthuong@gmail.com; 2 JMRC at Leibniz Institute for Natural Product Research and Infection Biology e.V. HKI and Friedrich Schiller University Jena, 07745 Jena, Germany; Kerstin.Voigt@hki-jena.de; 3 Departamento de Micologia, Federal University of Pernambuco, Av. da Engenharia, s/n, Recife PE 50740-600, Brazil; andrelcabral@msn.com; 4 Biodiversity Informatics and Spatial Analysis, Jodrell Laboratory, Royal Botanic Gardens Kew, Surrey TW9 3DS, UK; P.Kirk@kew.org

**Keywords:** 3 new taxa, ITS, LSU, Mucoromycota, phylogeny, taxonomy

## Abstract

Three novel fungal species, *Backusella chlamydospora* sp. nov., *B*. *koreana* sp. nov., and *B*. *thermophila* sp. nov., as well as two new records, *B*. *oblongielliptica* and *B*. *oblongispora*, were found in Cheongyang, Korea, during an investigation of fungal species from invertebrates and toads. All species are described here using morphological characters and sequence data from internal transcribed spacer sequences of ribosomal DNA and large subunit of the ribosomal DNA. *Backusella chlamydospora* is different from other *Backusella* species by producing chlamydospores. *Backusella koreana* can be distinguished from other *Backusella* species by producing abundant yeast-like cells. *Backusella* *thermophila* is characterized by a variable (subglobose to oblong, applanate to oval, conical and ellipsoidal to pyriform) columellae and grows well at 37 °C. Multigene phylogenetic analyses of the combined ITS and LSU rDNA sequences data generated from maximum likelihood and MrBayes analyses indicate that *B. chlamydospora*, *B*. *koreana*, and *B*. *thermophila* form distinct lineages in the family Backusellaceae. Detailed descriptions, illustrations, phylogenetic tree, and taxonomic key to the *Backusella* species present in Korea are provided.

## 1. Introduction

The genus *Backusella* (Mucoromycota, Mucorales) was established by Ellis and Hesseltine in 1969 [1] to describe *Mucor*-like species producing persistent-walled sporangiola on the lateral branches of their sporangiophores, which terminate in single deliquescent-walled sporangia; *Backusella circina* J.J. Ellis & Hesselt. is the type strain for this genus. Two years later, Pidoplichko and Milko [2] transferred *Thamnidium ctenidium* Durrell & M. Fleming to *Backusella* as *B*. *ctenidia* (Durrell & M. Fleming) Pidopl. & Milko and assigned both *B*. *circina* and *B*. *ctenidia* to the family Thamnidiaceae Fitzp. Benny and Benjamin [3] monographed the genus *Backusella*, leading to the definition of a new species, *B. lamprospora* (Lendn.) Benny & R.K. Benjamin (syn.: *Mucor lamprosporus* Lendn.). Between 1975 and 2013 the genus *Backusella* included only three species: *B*. *circina*, *B*. *lamprospora*, and *B*. *ctenidia*. However, Walther et al. [4] revised the Mucorales based on their ITS rDNA and LSU rDNA data and transferred all *Mucor* species with transitorily recurved sporangiophores to *Backusella*, while *B*. *ctenidia* was transferred to *Mucor*. This study expands the knowledge of genus *Backusella* to include nine species. Over the last few years, four new *Backusella* species were described based on phylogenetic and morphological analyses. The corresponding type strains were isolated in Brazil and Korea and named as follows: *B. azygospora* T.R.L. Cordeiro, Hyang B. Lee & A.L. Santiago [5], *B*. *constricta* D.X. Lima, C.A.F. de Souza & A.L. Santiago [6], *B*. *gigacellularis* J.I. de Souza, Piris-Zottar. & Harakava [7], and *B*. *locustae* Hyang B. Lee, S.H. Lee & T.T.T. Nguyen [8]. Recently, 10 new *Backusella* species were discovered in Australia by Urquhart et al. [9]—namely, *B. australiensis*, *B. liffmaniae*, *B. luteola*, *B. macrospora*, *B. mclennaniae*, *B. morwellensis*, *B. parvicylindrica*, *B. psychrophila*, *B. tarrabulga*, and *B. westeae*. Consequently, 25 species of *Backusella* are currently recorded in the Index Fungorum (www.indexfungorum.org; accessed 16 May 2021).

Voigt and Kirk (2012) established a family Backusellaceae to accommodate *Backusella*, based on morphological characters of sporangiophores and producing sporangiola in addition to sporangia in some species [10].

Members of *Backusella*, the unique genus of Backusellaceae, have been retrieved from soil, leaf litter, dung, moist wall, agarics, decaying wood, *Fragaria*, diseased roots, and *Medicago sativa* [3,4,5,6,7,8,9,11,12,13]. However, none of the *Backusella* species were isolated from invertebrates and toads. Thus, discovering new fungal species in specific and poorly studied substrates is pivotal for deepening our understanding of the biodiversity and phylogeny of *Backusella* as well as the actual diversity of mucoralean fungi. In Korea, only two species of this genus, *B*. *circina* and *B*. *locustae*, have been described [8,11]. Thus, many *Backusella* species still need to be investigated in Korea.

The aim of this study was to improve our understanding of the occurrence and distribution of *Backusella* species from Korea and to describe three new species. This is also the first report of *B*. *oblongielliptica* and *B*. *oblongispora* in Korea. A key to the *Backusella* species present in Korea is provided.

## 2. Materials and Methods

### 2.1. Sampling and Fungal Isolation

**Figure 1:**
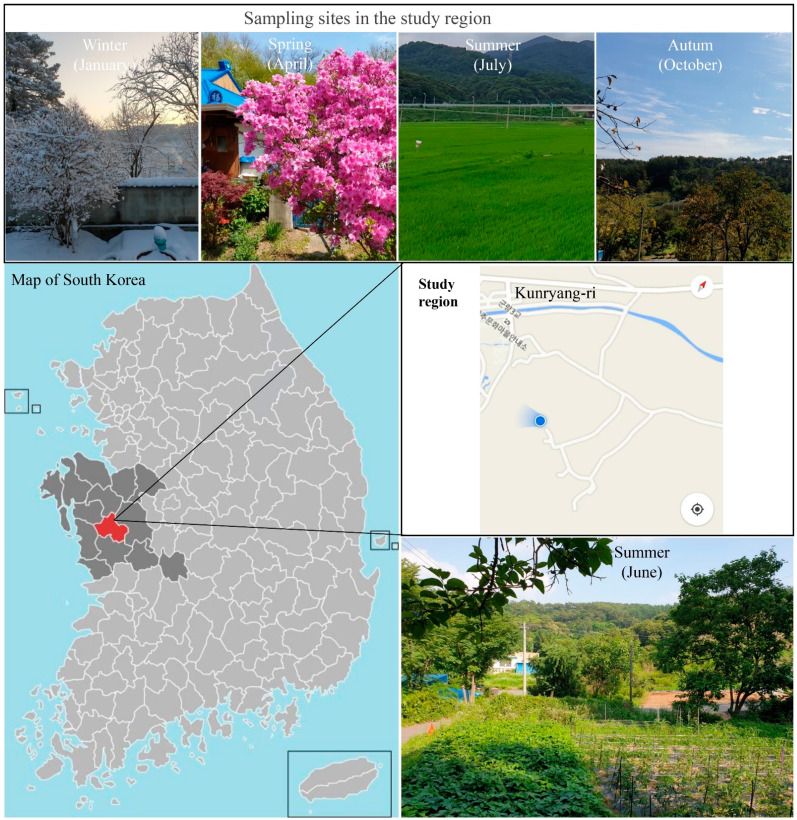
Map of sample collection sites. Samples were collected at Kunryang-ri, Cheongyang-eup, Cheongyang, Chungnam Province, Korea. Blue cursor represents the main area of Kunryang-ri for collection.

*Theuronema hilgendorfi hilgendorfi* (Scutigeromorpha, Scutigeridae), *Porcellio scaber* (Isopoda, Porcellinidae), *Timomenus komarovi* (Dermaptera, Forficulidae), *Gryllus bimaculatus* (Orthoptera, Gryllidae), *Scolopendra morsitans* (Scolopendromorpha, Scolopendridae), and *Bufo gargarizans* (Anura, Bufonidae) were collected at Kunryang-ri (36°44′00.2″ N 126°78′18.0″ E), Cheongyang, located in Chungnam Province, South Korea (Figure 1). The samples were captured and handled with gloved hands and were placed in polyethylene bags, Falcon conical tubes, and stored at ambient temperature until transported to the laboratory.

To isolate the fungal strains from *Bufo gargarizans*: the skin was swabbed with sterilized cotton swabs and then streaked on potato dextrose agar (PDA: Difco Laboratories, Detroit, MI, USA) with antibiotics (streptomycin sulfate 100 mg/L and penicillin 100 mg/L). The plates were then placed in the dark at 25 °C for 3–7 days. *Bufo gargarizans* were quickly released to the original site of collection after isolation of fungi. 

To isolate the fungal strains from *Theuronema hilgendorfi hilgendorfi*, *Porcellio scaber*, *Timomenus komarovi*, *Gryllus bimaculatus*, and *Scolopendra morsitans*: samples were removed from the bags or Falcon tubes and transferred to clean Petri dishes. Bodies were then broken up into small pieces and placed on PDA. The plates were then placed in the dark at 25 °C and checked under a stereomicroscope every day after the second day of incubation. Then, hyphal tips were transferred to fresh PDA. Single-spore cultures were established as described by Choi et al. [14]. Briefly, spores from a single sporangium after 2–4 days incubation were transferred to sterilized water using capillary tubes and then vortexed to obtain a homogeneous spore suspension. Spore suspensions were diluted several times (1:10) and 50 μL of each dilution was spread on PDA medium. The plates were then incubated at 25 °C to allow for spore germination. Colonies from single spores were transferred to a new PDA plate and used for DNA extraction. Ex-type living cultures were deposited at Chonnam National University Fungal Collection, Gwangju, South Korea (CNUFC), Gwangju, Korea. Dried cultures were deposited in the Herbarium Chonnam National University, Gwangju, Korea.

### 2.2. Morphological Studies

Pure cultures of *Backusella* spp. were grown on synthetic mucor agar (SMA: 40 g dextrose, 2 g asparagine, 0.5 g KH_2_PO_4_, 0.25 g MgSO_4_·7H_2_O, 0.5 mg thiamine hydrochloride, and 15 g agar in 1 L of deionized water), PDA, and malt extract agar (MEA: 40 g malt extract, 4 g yeast extract, and 15 g agar in 1 L of deionized water). Plates were incubated at 25 °C in the dark for 5 to 14 days. Cultures grown on SMA at 25 °C were photographed, and the dimensions of sporangia, sporangiola, columellae, sporangiospores, and chlamydospores were measured. Fragments of mycelia were removed from each culture and placed onto microscope slides with 60% lactic acid. A differential interference contrast microscope (Olympus BX51, Olympus, Tokyo, Japan) was used to generate digital images. For temperature studies, the isolates were grown in triplicates on SMA and were incubated at 10, 20, 25, 30, 37, or 40 °C. Colony growth was measured every 24 h for 7 days.

### 2.3. DNA Extraction, PCR, Cloning, and Sequencing

*Backusella* spp. were cultured on cellophane over PDA at 25 °C for 3 days. The mycelial mass was collected by scraping from the surface of cellophane and was placed in 1.5 mL sterile Eppendorf tubes. Genomic DNA was then extracted using the SolgTM Genomic DNA Preparation Kit (Solgent Co. Ltd., Daejeon, Korea) according to the manufacturer’s protocol. The purified DNA was stored at −20 °C for later use. Two regions were amplified: the internal transcribed spacer (ITS) region, using the primers V9G/ITS 4 [15,16] and ITS1/ITS4 [16]; and the large subunit (LSU) rRNA gene region, using the primers LROR [17] and LR7 [18]. These target regions, the ITS, and the LSU rRNA gene, were amplified by PCR in 20 μL reaction mixtures containing 2 μL of genomic DNA, 1.5 μL of each primer (5 pM), 14 μL of sterile water, and 1 μL of PCR premix (Bioneer, Daejeon, Korea). Reaction mixtures were pre-heated at 95 °C for 4 min, and PCR was performed as follows: 30 cycles of 45 s at 95 °C, 30 s at 52 °C, and 45 s at 72 °C, with a final extension at 72 °C for 7 min, followed by cooling at 4 °C. PCR products were visualized in 1% (*w/v*) agarose gel electrophoresis. The PCR products were then purified using an Accuprep PCR Purification Kit (Bioneer) and sequenced on the ABI PRISM 3730XL Genetic Analyzer (Applied Biosystems, Foster City, CA, USA) using the same primers employed for PCR. In some cases, it is necessary to clone the ITS sequences before sequencing. PCR products were cloned using the pGEM-T Easy Vector System cloning kit (Promega, Madison, WI, USA), following the manufacturer’s instructions. These clones were sequenced using the primers M13F forward (5′-GTAAAACGACGGCCAGT-3′) and M13R-pUC reverse (5′-CAGGAAACAGCTATGAC-3′).

### 2.4. Sequence Alignment and Phylogenetic Analyses

Each generated sequence was checked for the presence of ambiguous bases and was assembled using Lasergene SeqMan program from DNASTAR, Inc. (Madison, WI, USA). Edited sequences were blasted against the NCBI GenBank nucleotide database (https://blast.ncbi.nlm.nih.gov/Blast.cgi; 8 September 2020) to search for closest relatives. Sequences of all the accepted *Backusella* species according to the last update of the Index Fungorum database were retrieved from GenBank. Sequences were aligned using MAFFT (http://mafft.cbrc.jp/alignment/server; 15 January 2021) with the algorithm L-INS-I. Aligned sequences were automatically trimmed using trimAl [19] with the gappyout method. Data were converted from a fasta format to nexus and phylip formats using the online tool Alignment Transformation Environment (https://sing.ei.uvigo.es/ALTER/; 15 January 2021) [20]. Phylogenetic reconstructions by maximum likelihood (ML) and Bayesian inference (BI) were carried out using PhyML 3.0 [21] and MrBayes 3.2.2 [22], respectively. We performed the ML analysis using 1000 bootstrap replicates. BI analyses were performed using three million Markov chain Monte Carlo (MCMC) generations. The sample frequency was set to 100, and the first 25% of trees were removed as burn-in. The best substitution models for each data partition were estimated using jModelTest v.2.1.10 according to the Akaike criterion [23,24]. The newly obtained sequences were deposited in the GenBank database under the accession numbers provided in Table 1.

## 3. Results

### 3.1. Phylogenetic Analysis

**Figure 2:**
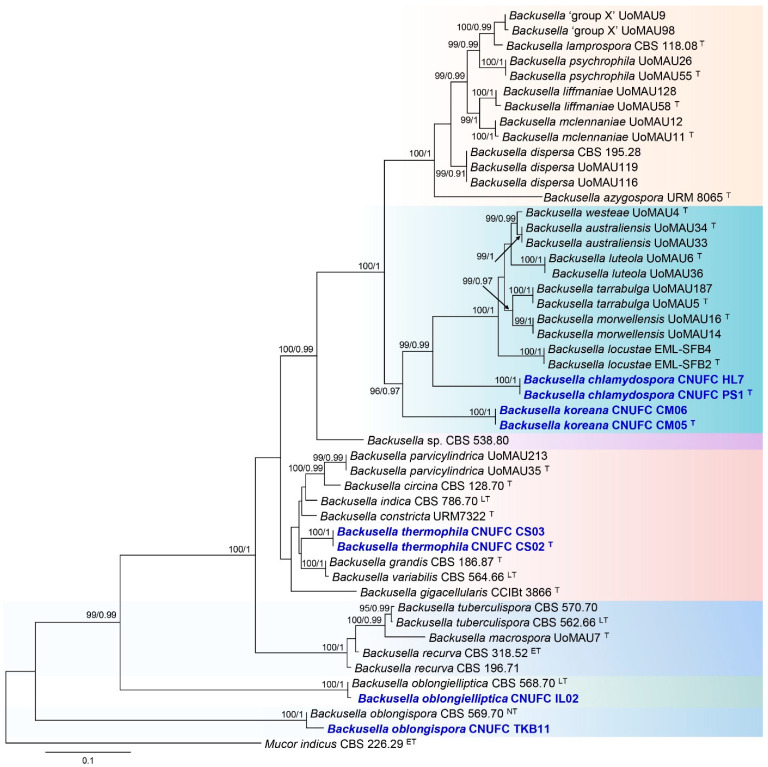
Phylogenetic tree constructed by maximum likelihood analysis of 49 sequences of the combined ITS and LSU. The numbers above branches represent maximum likelihood bootstrap percentages (left) and Bayesian posterior probabilities (right). Bootstrap values ≥70% and Bayesian posterior probabilities ≥0.90 are shown. The bar indicates the number of substitutions per position. *Mucor indicus* CBS 226.29 was used as outgroup. Ex-type, ex-epitype, ex-lectotype, and ex-neotype strains are marked with ^T^, ^ET, LT^, and ^NT^, respectively. Newly generated sequences are in bold blue.

The phylogenetic relationship of the new *Backusella* species with accepted species was determined by analysis of concatenated sequence datasets of two loci (ITS and LSU). The combined ITS and LSU sequence dataset consisted of 49 taxa, and the aligned dataset was comprised of 1120 characters including gaps (ITS: 1–530 and LSU: 531–1120). The BI and ML analyses of this dataset were based on the GTR + I + G model. The trees generated from Bayesian analyses showed the similar topologies (data not shown here). Therefore, we only present the tree obtained from ML analysis (Figure 2).

The two new species, CNUFC PS1 and CNUFC CM05, are close to *B*. *locustae*, *B*. *morwellensis*, *B*. *tarrabulga*, *B*. *australiensis*, *B*. *westeae*, and *B*. *luteola*, and they form a separate lineage based on multi-loci gene phylogeny. The new species CNUFC CS02 is close to *B*. *constricta*, *B*. *grandis*, and *B*. *variabilis*, and it forms a separate lineage, while strains CNUFC IL02 and CNUFC TKB11 group together with reported species *B. oblongielliptica* and *B*. *oblongispora*, respectively, in a monophyletic clade (Figure 2).

### 3.2. Taxonomy

Our analysis revealed the presence of three new species and two new records in genus *Backusella*. These species are described below.

**Figure 3:**
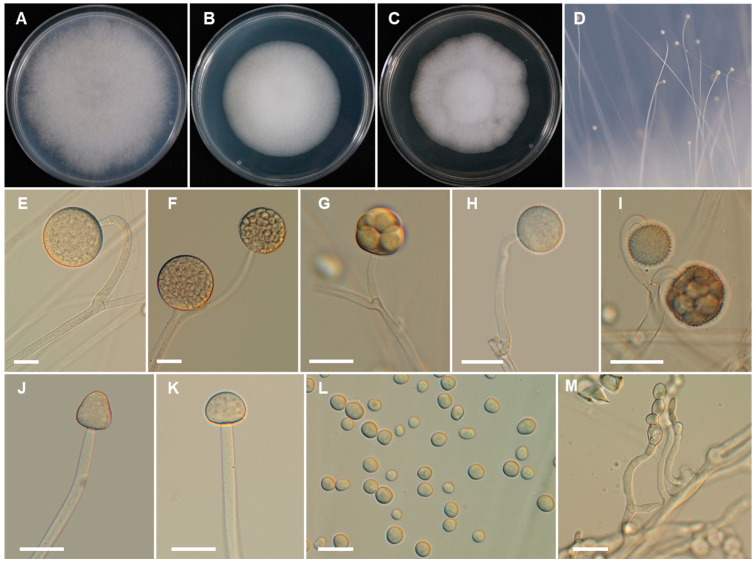
Morphology of *Backusella chlamydospora*. (**A**), Colony on SMA. **B**), Colony on PDA. (**C**), Colony on MEA. (**D**), Sporangiophores forming sporangia. (**E**,**F**), Sporangia. (**G**), Multi-spored sporangiolum. (**H**), Uni-spored sporangiolum. (**I**), Uni- and multi-spored sporangiola borne on circinate branches. (**J**,**K**), Columellae of different shapes. (**L**), Sporangiospores. (**M**), Chlamydospores. Scale bars = 20 μm.

*Backusella chlamydospora* Hyang B. Lee & T.T.T. Nguyen sp. nov. (Figure 3).

Index Fungorum: IF554922.

*Etymology*: Referring to the production of chlamydospores.

*Type specimen:* REPUBLIC OF KOREA, in a house garden located on a hill in Kunryang-ri (36°44′00.2″ N, 126°78′18.0″ E), Cheongyang-eup, Cheongyang, Chungnam Province, from the head of a *Porcellio scaber*, 20 April 2020, H.B. Lee (holotype CNUFC HT2018; cultures ex-type CNUFC PS1).

*Description*: Colonies develop rapidly on SMA, reaching a diameter of 62 mm after 4 days of incubation at 25 °C. Colonies white at first, become light gray with age; reverse light gray. Sporangiophores arising from the substrate mycelia curved when young and erect at maturity, with or without yellow granular content, frequently broaden upwards and then tapering slightly toward the sporangium, at first simple and later bearing pedicellate multispored or unispored sporangiola, 6.0–12.5 μm diameter. Lateral circinate branches simple or rebranched: simple branches ending in a sporangium, multi-or uni-spored sporangiola, and complex branches ending in sporangiola, sometimes bearing a sterile sporangium. Sporangia globose to subglobose, multi-spored, with spinulose and deliquescent wall, yellowish but later turning brown, 35–80 × 35–75 μm. Columellae of sporangia variable in shape (subglobose, conical, ellipsoidal, cylindrical, hemi-spherical, or near pyriform; sometimes bell-shaped, long conical, or constricted at the middle portion), smooth, with or without yellowish granular contents, 18.5–35 × 15.5–30 μm. Collar present. Multi-spored sporangiola contain (2–)3–18(–26) sporangiospores, globose to subglobose, (15–)17.5–35(–37) × (15–)17–33(–35) μm, wall persistent, and spinulose. More abundant production of multi-spored sporangiola was observed after 8 days of culture. The columellae of sporangiola subglobose, or conical, up to 12.5 μm diameter, smooth walled. Uni-spored sporangiola abundant near the substrate, globose, (10.5–)13.5–23(–30) μm diameter, wall persistent, and spinulose. The sporangiospores of sporangia and multi-spored sporangiola are similar: mostly subglobose or, less frequently, globose or irregular in shape, (6.5–)7.5–13.5(–15) × (6–)6.5–12.5(–13.5) µm. Sporangiospores from multi-spored sporangiola up to 20.5 × 17.5 µm in older cultures (15 days). Substrate mycelia highly branched, containing inflated regions ending in thin rhizoid-like filaments with green droplets. Chlamydospores produced after 4 days at the ends of vegetative hyphae, oidium-like, thick walled, terminal or in chains of 2 to 19 spores, globose to subglobose, 7.5–14.5 × 6.5–12 µm, or ellipsoidal or cylindrical to irregular, 9.5–23 × 7.5–13.5 µm, thick-walled. Zygosporangia not observed.

*Culture characteristics*: Colony diam, 72 h, in mm: SMA 25 °C 42; SMA 20 °C 33; SMA 30 °C 30; SMA 37 °C no growth; MEA 25 °C 39; PDA 25 °C 34.

*Additional material examined*: REPUBLIC OF KOREA, in a house garden located on a hill in Kunryang-ri (36°44′00.2″ N, 126°78′18.0″ E), Cheongyang-eup, Cheongyang, Chungnam Province, from the middle part of a *Theuronema hilgendorfi hilgendorfi*, 17 July 2020, H.B Lee (culture CNUFC HL7).

**Figure 4:**
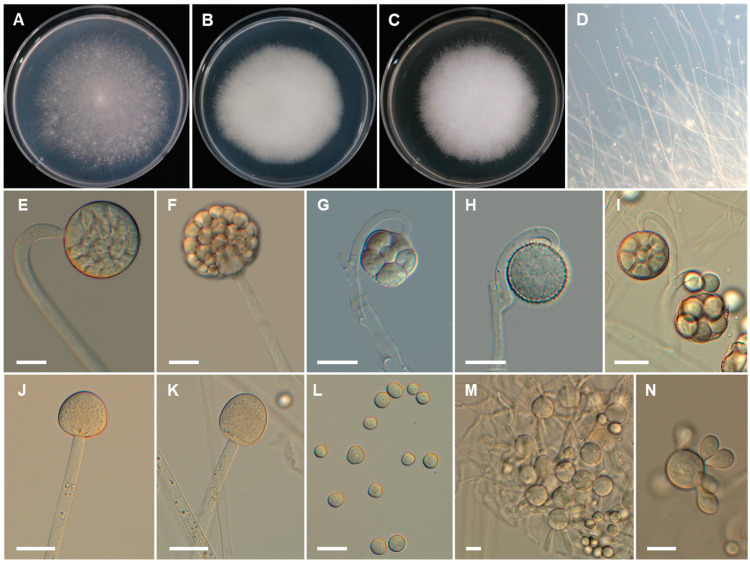
Morphology of *Backusella koreana*. (**A**), Colony on SMA. (**B**), Colony on PDA. (**C**), Colony on MEA. (**D**), Sporangiophores forming sporangia. (**E**,**F**), Sporangia. (**G**), Multi-spored sporangiolum. (**H**), Uni-spored sporangiolum. (**I**), Multi-spored sporangiola borne on circinate branches. (**J**,**K**), Columellae of different shapes. (**L**), Sporangiospores. (**M**,**N**), Yeast-like cells. Scale bars = 20 μm.

*Backusella koreana* Hyang B. Lee, J.S. Kim & T.T.T. Nguyen, sp. nov. (Figure 4). 

Index Fungorum: IF554923.

*Etymology*. Referring to the country in which it was isolated.

*Type specimen:* REPUBLIC OF KOREA, in a house garden located on a hill of Kunryang-ri (36°44′00.2″ N, 126°78′18.0″ E), Cheongyang-eup, Cheongyang, Chungnam Province, from the head of *Scolopendra morsitans*, 21 June 2020, H.B. Lee and J.S. Kim (holotype CNUFC HT2020; ex-type living culture, CNUFC CM05).

*Description*: Colonies on SMA developing rapidly, reaching 65 mm diameter after 4 days of incubation at 25 °C. Colonies white at first, becoming light gray when old; reverse light gray. Sporangiophores arising from substrate mycelia, curved when young and erect at maturity, with or without yellow granular content, frequently broader upwards and then tapering slightly toward the sporangium, at first simple, later bearing pedicellate multispored or unispored sporangiola, 6.5–15(–20) μm wide. Lateral circinate branches simple or rebranched: simple branches end in sporangia, or multi or uni-spored sporangiola; branches ending in sporangiola sometimes bearing a sterile sporangium. Sporangia yellowish but later turning brown, globose to subglobose, multi-spored, (31–)40–70(–85) × 30–70(–80) μm, wall spinulose and deliquescent, yellowish but later turning brown. Columellae of sporangia globose to ellipsoidal, sometimes hemi-spherical, with or without yellowish granular contents, 20–30 × 15–30 μm. Collar present. Multi-spored sporangiola production observed after 7 days of culture. Multi-spored sporangiola contain (2–)3–8(–10) sporangiospores, globose to subglobose, (13–)16–35(–40) × (12–)15–32(–40) μm, wall spinulose and persistent. Columellae of sporangiola subglobose (up to 17 μm long), or conical (up to 13 μm long), smooth walled. Unispored sporangiola rare, globose, 15–30 μm diameter, wall spinulose and persistent. Sporangiospores of sporangia and multi-spored sporangiola are similar, mostly subglobose or globose or irregular in shape, 10–18 × 10–17 µm. Substrate highly branched, containing inflated regions terminating in thin rhizoid-like filaments and green droplets. Yeast-like cells were abundant on SMA, globose to oval. Chlamydospores and zygospores not observed.

*Culture characteristics*: Colony diam, 72 h, in mm: SMA 25 °C 48; SMA 20 °C 33; SMA 30 °C 30; SMA 10 °C 4; SMA 37 °C no growth; MEA 25 °C 44; PDA 25 °C 46.

*Additional material examined*: REPUBLIC OF KOREA, in a house garden located on a hill of Kunryang-ri (36°44′00.2″ N, 126°78′18.0″ E), Cheongyang-eup, Cheongyang, Chungnam Province, from the head of a *Scolopendra morsitans*, 22 June 2020, H.B. Lee and J.S. Kim (culture CNUFC CM06).

**Figure 5:**
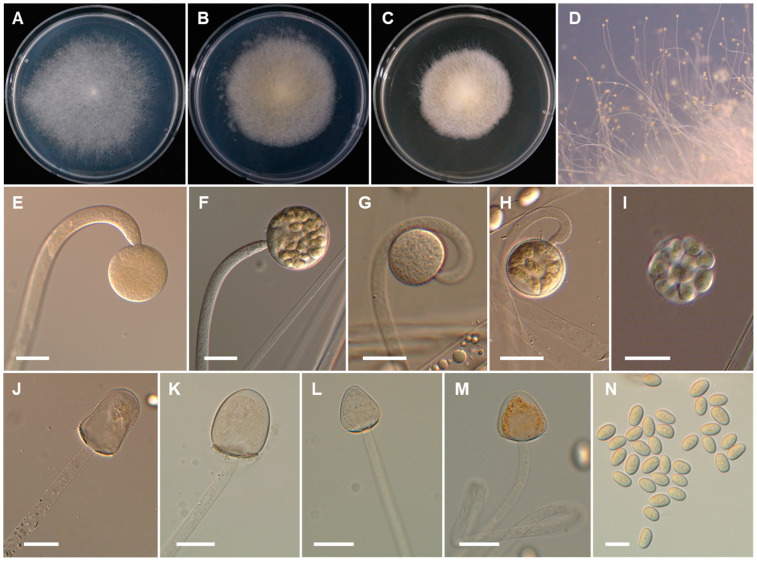
Morphology of *Backusella thermophila*. (**A**), Colony on SMA. (**B**), Colony on PDA. (**C**), Colony on MEA. (**D**), Sporangiophores forming sporangia. (****E–**H**), Young and mature sporangia. (**I**), Multi-spored sporangiolum. (**J**–**M**), Columellae of various shapes. (**N**), Sporangiospores. Scale bars = 20 μm.

*Backusella thermophila* Hyang B. Lee, A.L. Santiago, P.M. Kirk, K. Voigt & T.T.T. Nguyen sp. nov. (Figure 5).

Index Fungorum: IF554924.

*Etymology*: Refers to the ability to grow and sporulate at high temperature (>37 °C).

*Type specimen:* REPUBLIC OF KOREA, in a house garden located on a hill of Kunryang-ri (36°44′00.2″ N, 126°78′18.0″ E), Cheongyang-eup, Cheongyang, Chungnam Province, from the leg of *Gryllus bimaculatus*, on 27 July 2020, H.B. Lee (holotype, CNUFC HT2019; ex-type living culture, CNUFC CS02).

*Description*: Colonies on SMA developing rapidly, reaching 68 mm diameter after 4 days incubation at 25 °C. Colonies white at first, becoming light yellow when old; reverse light yellow. Sporangiophores arising from substrate mycelia curved when young and erect at maturity, 7.5–25 µm; unbranched or, less frequently, with a short, recurved branch bearing a sterile sporangium, which quickly reaches the lid of the Petri dish. Sporangia yellow, deliquescent, globose to subglobose, (35–)40–95(–130) µm diameter, wall deliquescent, transparent, and spinulose, with spines 3.5–7.5 µm long. Columellae subglobose to oblong (35–80 × 30–70 µm), applanate to oval (22–40 × 20–30 µm), or conical or ellipsoidal to pyriform (42–66 × 33–48 µm), with or without brownish contents. Collars small. Multi-spored sporangiola production observed after 7 days; sporangiola contain (4–)8–18 sporangiospores, globose to subglobose, 20–40 μm diameter, wall persistent and spinulose. Columellae of sporangiola subglobose or conical, 15–17 × 10–15 μm, smooth walled. Uni-spored sporangiola rare, globose, up to 15 μm diameter, spinulose. Sporangiospores of sporangia and multi-spored sporangiola mostly ellipsoidal, 14–20 × 10–13 µm, yellowish or with yellow granules concentrated in the center. Substrate highly branched, containing inflated regions, and terminating in thin rhizoid-like filaments. Chlamydospores and zygospores not observed. 

*Culture characteristics*: Colony diam, 72 h, in mm: SMA 25 °C 42; SMA 20 °C 33; SMA 30 °C 45; SMA 10 °C 3; SMA 37 °C 38; MEA 25 °C 39; PDA 25 °C 43.

*Additional materials examined*: REPUBLIC OF KOREA, in a house garden located on a hill in Kunryang-ri (36°44′00.2″ N, 126°78′18.0″ E), Cheongyang-eup, Cheongyang, Chungnam Province, from the leg of *Gryllus bimaculatus*, 28 July 2020, H.B. Lee (culture CNUFC CS03).

**Figure 6:**
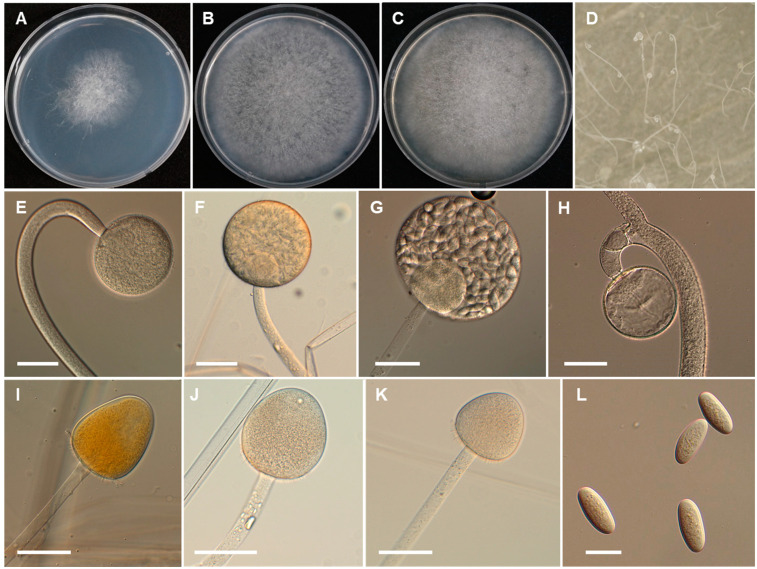
Morphology of *Backusella oblongielliptica*. (**A**), Colony on SMA. (**B**), Colony on PDA. (**C**), Colony on MEA. (**D**), Sporangium on circinate branches. (**E**–**G**), Young and mature sporangia. (**H**), Sterile sporangium. (**I**–**K**), Columellae of different shapes. (**L**), Sporangiospores. Scale bars: E–K = 50 μm, L = 20 μm.

*Backusella oblongielliptica* (H. Nagan., Hirahara & Seshita ex Pidopl. & Milko) G. Walther & de Hoog, Persoonia 30: 41 (2013) (Figure 6).

Basionym. *Mucor oblongiellipticus* H. Nagan., Hirahara & Seshita ex Pidopl. & Milko, Atlas Mukor. Grib. (Kiev): 81 (1971).

≡ *Mucor oblongiellipticus* H. Nagan., Hirahara & Seshita, Essays Stud. Fac. Hiroshima Jogakuin College 18: 167 (1969), nom. inval., Art. 36.1.

*Description*: Colonies on SMA developing rapidly, reaching 72 mm diameter after 4 days incubation at 25 °C. Colonies white; reverse white. Sporangiophores arising from substrate mycelia, curved when young and erect at maturity, 15–35 µm wide, unbranched or branched at the base. Sporangia yellow, exhibiting or not yellowish- or reddish-brown contents, globose to subglobose, 65–195(–215) × 60–180(–210) µm, yellow, wall transparent and deliquescent. Columellae subglobose to ellipsoidal, (35–)40–90(–115) × (35–)40–80(–110) µm. Collar present, some with needle-shaped spines. Sporangiospores oblong to ellipsoidal, (28–)30–40 × 12–15(–18) µm, yellowish or with yellow granules concentrated in the center. Substrate mycelia highly branched. Sporangiola not formed. Chlamydospores, and zygospores not observed.

*Culture characteristics*: Colony diam, 72 h, in mm: SMA 25 °C 54; SMA 20 °C 59; SMA 30 °C 46; SMA 10 °C 13; SMA 37 °C no growth; MEA 25 °C 65; PDA 25 °C 70.

*Specimen examined*: South Korea, in a house garden located on a hill of Kunryang-ri (36°44′00.2″ N, 126°78′18.0″ E), Cheongyang-eup, Cheongyang, Chungnam Province, from the middle part of a *Timomenus komarovi*, 14 April 2020, J.S. Kim (culture CNUFC IL02).

**Figure 7:**
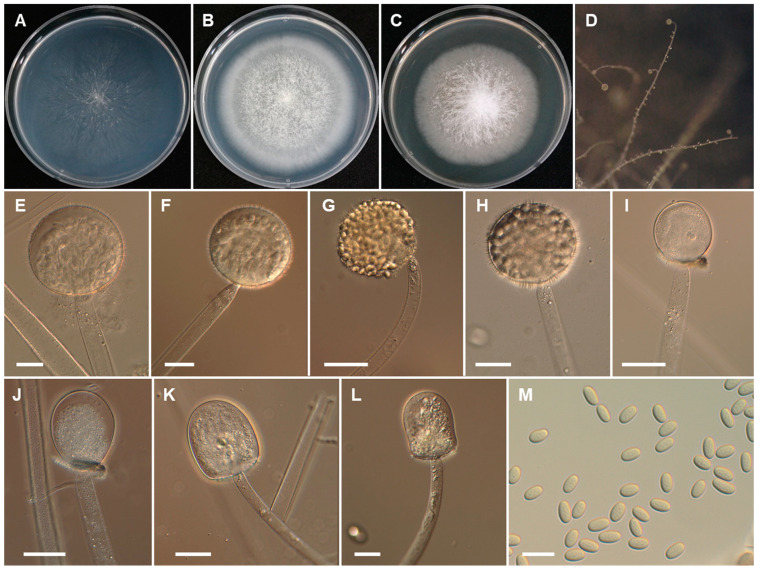
Morphology of *Backusella oblongispora*. (**A**), Colony on SMA. (**B**), Colony on PDA. (**C**), Colony on MEA. (**D**), Sporangium on circinate branches. (**E**–**H**), Young and mature sporangia. (**I**–**L**), Columellae of various shapes. (**M**), Sporangiospores. Scale bars: E–L = 50 μm, M = 20 μm.

*Backusella oblongispora* (Naumov) G. Walther & de Hoog, Persoonia 30: 41 (2013) (Figure 7).

Basionym. *Mucor oblongisporus* Naumov, Mater. Mykol. Fitopatol. Rossii 1(4): 12 (1915).

*Description*: Colonies growing fast on SMA, reaching 58 mm diameter after 4 days incubation at 25 °C. Colonies white at first, becoming smoke gray when old; reverse white. Sporangiophores arising from substrate mycelium, curved when young, erect at maturity. Sporangiophores, 12–42 µm wide, displaying a wider base and a slight constriction next to the sporangium, sporangiophores at first curved, unbranched, but later branched, sometimes bearing a sterile sporangium. Sporangia produced after 86 h, yellowish, globose to subglobose, (40–)70–170(–265) × (40–)65–165(–260) µm, finely echinulate. Columellae exhibiting or not brownish contents, ellipsoidal to pyriform or oblong, 50–115(–160) × 40–80(–130) µm. Collar present, some with needle-shaped spines. Sporangiospores ellipsoidal, 8.5–12 × 14–18 µm, smooth-walled. Sporangiola not formed. Chlamydospores and zygospores not observed.

*Culture characteristics*: Colony diam, 72 h, in mm: SMA 25 °C 44; SMA 20 °C 38; SMA 30 °C 36; SMA 10 °C 17; SMA 37 °C no growth; MEA 25 °C 43; PDA 25 °C 53.

*Specimen examined*: South Korea, in a house garden located on a hill in Kunryang-ri (36°44′00.2″ N, 126°78′18.0″ E), Cheongyang-eup, Cheongyang, Chungnam Province, on skin of *Bufo gargarizans*, 11 June 2020, J.S. Kim (culture CNUFC TKB11).

## 4. Discussion

Cheongyang, located in Chungnam Province, South Korea, covers a total area of 479.7 square kilometers, about 65.8% of which is forest, 7.3% field, and 14.7% paddy field. It is also known for the spicy gochu peppers that are produced in the region and for the fruit of the Chinese matrimony vine, or gugija, which make Cheongyang a favorable region for the growth of fungi. This area is more recognized as a hotspot for biodiversity and is known as the “Alps of Chungnam” and a place that needs to be preserved. In 2019, we found a new species of *Mucor*, *M*. *cheongyangensis* from the surface of *Lycorma delicatula* in this region [25]. Here, three new species to genus *Backusella* from the Cheongyang area are described and are compared with those of their most closely related species.

Our phylogenetic analyses showed that *B*. *koreana* and *B*. *chlamydospora* form a separate lineage, phylogenetically related to *B*. *locustae*, *B*. *morwellensis*, *B*. *tarrabulga*, *B*. *australiensis*, *B*. *westeae*, and *B*. *luteola*. However, the production of chlamydospores can be used to easily distinguish *B*. c*hlamydospora* from these species. In addition, *B*. *chlamydospora* produces larger sporangia than *B*. *luteola* (26–59 × 23–55 µm), *B*. *liffmaniae* (26–55 × 26–54 µm), *B*. *morwellensis* (23–72 × 23–57 µm), and *B*. *westeae* (26–64 × 22–58 µm) [9]. As observed in *B*. *chlamydospora*, *B. constricta*, *B*. *lamprospora*, *B*. *circina*, and *B*. *azygospora* also produce both uni-spored and multi-spored sporangiola. Moreover, *B*. *chlamydospora* can grow at 35 °C, while *B*. *azygospora* is unable to grow at this temperature [5]. In addition, azygospores are quite common in the latter but not produced by the former. Like *B*. *chlamydospora*, *B. circina* and *B*. *constricta* also produce subglobose sporangiospores, but pyriform columellae have not been observed in either of these species. In addition, *B. chlamydospora* produces a higher number of spores per multi-spored sporangiola than *B*. *constricta* (2–8) and *B*. *circina* (2–14) [1,6]. *Backusella koreana* is a sister taxon to *B*. *chlamydospora*. Both morphological and phylogenetic analyses indicate differences between these two new taxa. For example, *B. chlamydospora* produces a larger number of spores on multi-spored sporangiola than *B*. *koreana.* Furthermore, the sporangiospores of *B*. *koreana* are larger than those of *B*. *chlamydospora*. In addition, *B*. *chlamydospora* can be distinguished from *B*. *koreana* by production of chlamydospores. Finally, *B. koreana* produces abundant yeast-like cells on SMA medium. *Backusella thermophila* is phylogenetically related to *B*. *constricta*, *B*. *grandis*, and *B*. *variabilis*. Morphologically, the new species can be easily distinguished from *B. constricta* by the size and shape of sporangiospores that are subglobose to elliptical, some slightly irregular in shape, with 7.5–15 × 5.5–10 μm in diameter in the later species [6]. In addition, *B. constricta* displayed limited growth at 37 °C, but *B*. *thermophila* was able to grow well even at 37 °C. Finally, *B. constricta* produces some constrict columellae, which are rarely observed in *B*. *thermophila*. In fact, *B*. *thermophila* produces columellae varied in shape (subglobose to oblong, applanate to oval, conical and ellipsoidal to pyriform), whereas *B*. *grandis* produces conical to cylindrical-ellipsoidal columellae [4,26]. *Backusella grandis* differs from *B. thermophila* by the production of bigger columella (115–200 × 100–180 µm) [4,26]. *Backusella thermophila* and *B*. *variabilis* grow at 37 °C; however, *B. thermophila* differs from *B*. *variabilis* by having smaller sporangia and producing both multispored and uni-spored sporangiola [4,27]. Therefore, morphological analysis supported the results of molecular phylogenetic analysis, indicating that these new isolates are new species described here as *B*. *chlamydospora*, *B. koreana*, and *B*. *thermophila*.

Most species of *Backusella* are saprobic and commonly isolated from soil and leaf litter [4,9]. Some species have been found in excrement of humans, herbivores, or insects [4,8,13]. However, the genus *Backusella* has not been involved in human infections [28]. Interestingly, the new species (*B*. *chlamydospora*, *B. koreana*, and *B*. *thermophila*), and two newly recorded species (*B. oblongielliptica* and *B*. *oblongispora*) of *Backusella* were first discovered from invertebrate and toad samples. The presence of *Backsuella* species in different substrates reflects their ecological importance in ecosystems. Further studies on the fungal diversity of the niches are needed to understand the relationships between these organisms in ecosystems.

The identification of these novel species expands the range of potential habitats of the members of this genus. This suggests that unknown micro-fungi, especially those belonging to the genus *Backusella,* have not yet been described and that invertebrate samples may be a rich source of novel species of fungi.

**Key to *Backusella* species in Korea**

1. Chlamydospores present……………………………………………. *B*. *chlamydospora*

1. Chlamydospores absent……………………………………………………………….. 2

2. Yeast-like cells present ……………………………………………………… *B*. *koreana*

2. Yeast-like cells not observed………………………………………………………….. 3

3. Sporangia commonly bigger than 150 µm diam., sporangiola not formed……… 5

3. Sporangia up to 130 µm diam., sporangiola formed……………………………….. 4

4. Unispored sporangiola abundant ……………………………………………*B*. *circina*

4. Unispored sporangiola rarely present, or not observed ………………................... 6

5. Columellae subglobose to ellipsoidal; sporangiospores oblong to ellipsoidal, (28–)30–40 × 12–15(–18) µm………………………………………………*B*. *oblongielliptica*

5. Columellae ellipsoidal to pyriform; sporangiospores ellipsoidal, 8.5–12 × 14–18 µm……………………………………………………………………............ *B*. *oblongispora*

6. Sporangiospores globose to subglobose; no growth at 37 °C ……………. *B*. *locustae*

6. Sporangiospores ellipsoidal; good growth and sporulation at 37 °C … *B*. *thermophila*

## Figures and Tables

**Table 1:** Taxon, collection number, sequence, and GenBank accession number relative to all ITS and LSU sequences used in this study.

Taxon Name	Strain Number	Host/Substrate	Country	GenBank Accession Number	
				ITS	LSU
*B. australiensis*	UoMAU34 ^T^	Leaf litter	Australia	MK959062	MK958800
*B. australiensis*	UoMAU33	–	Australia	–	MK958801
*B. azygospora*	URM 8065 ^T^	Soil	Brazil	MK625216	MK625222
***B. chlamydospora***	**CNUFC PS1 ^T^**	***Porcellio scaber***	**South Korea**	**MZ171385**	**MZ148709**
***B. chlamydospora***	**CNUFC HL7**	***Theuronema hilgendorfi hilgendorfi***	**South Korea**	**MZ171386**	**MZ148710**
*B. circina*	CBS 128.70 ^T^	Soil with lichens	USA	JN206258	JN206529
*B. constricta*	URM 7322 ^T^	Soil	Brazil	KT937158	KT937156
*B. dispersa*	CBS 195.28	Fallen leaf	USA	JN206271	JN206530
*B. dispersa*	UoMAU119	–	Australia	–	MK958770
*B. dispersa*	UoMAU116	–	Australia	–	MK958769
*B. gigacellularis*	CCIBt 3866 ^T^	Leaf litter canopy-plates	Brazil	KF742415	–
*B. grandis*	CBS 186.87 ^T^	Dung of mouse	India	JN206252	JN206527
*B. indica*	CBS 786.70 ^LT^	–	India	JN206255	JN206526
***B. koreana***	**CNUFC CM05 ^T^**	***Scolopendra morsitans***	**South Korea**	**MZ171387**	**MZ148711**
***B. koreana***	**CNUFC CM06**	***Scolopendra morsitans***	**South Korea**	**MZ171388**	**MZ148712**
*B. lamprospora*	CBS 118.08 ^T^	–	Switzerland	JN206268	JN206531
*B. liffmaniae*	UoMAU58 ^T^	Leaf litter	Australia	MK959065	MK958734
*B. liffmaniae*	UoMAU128	–	Australia	–	MK958735
*B. locustae*	EML-SFB4	Grasshopper feces	South Korea	KY449293	KY449290
*B. locustae*	EML-SFB2 ^T^	Grasshopper feces	South Korea	KY449291	KY449292
*B. luteola*	UoMAU6 ^T^	Leaf litter	Australia	MK959058	MK958795
*B. luteola*	UoMAU36	–	Australia	–	MK958794
*B. macrospora*	UoMAU7 ^T^	Leaf litter	Australia	MK959107	MK958628
*B. mclennaniae*	UoMAU11 ^T^	Leaf litter	Australia	MK958776	MK958776
*B. mclennaniae*	UoMAU12	–	Australia	MK959087	MK958777
*B. morwellensis*	UoMAU16 ^T^	Leaf litter	Australia	MK959059	MK958808
*B. morwellensis*	UoMAU14	–	Australia	–	MK958806
*B. oblongielliptica*	CBS 568.70 ^LT^	Agaric	Japan	JN206278	JN206533
***B. oblongielliptica***	**CNUFC IL02**	***Timomenus komarovi***	**South Korea**	**MZ171391**	**MZ148715**
*B. oblongispora*	CBS 569.70 ^NT^	Soil	Japan	JN206251	JN206407
***B. oblongispora***	**CNUFC TKB11**	**Skin of *Bufo gargarizans***	**South Korea**	**MZ420786**	**MZ148717**
*B. parvicylindrica*	UoMAU35 ^T^	Leaf litter	Australia	MK959109	MK958727
*B. parvicylindrica*	UoMAU213	–	Australia	–	MK958732
*B. psychrophila*	UoMAU55 ^T^	Leaf litter	Australia	MK959093	MK958749
*B. psychrophila*	UoMAU26	–	Australia	–	MK958748
*B. recurva*	CBS 318.52 ^ET^	Fragaria; diseased root	USA	JN206261	JN206522
*B. recurva*	CBS 196.71	–	n.a	JN206265	JN206523
*Backusella* sp.	CBS 538.80	*Medicago sativa*	Egypt	HM999964	HM849692
*B. tarrabulga*	UoMAU5 ^T^	Leaf litter	Australia	MK959060	MK958804
*B. tarrabulga*	UoMAU187	–	Australia	–	MK958805
***B. thermophila***	**CNUFC CS02 ^T^**	***Gryllus bimaculatus***	**South Korea**	**MZ171389**	**MZ148713**
***B. thermophila***	**CNUFC CS03**	***Gryllus bimaculatus***	**South Korea**	**MZ171390**	**MZ148714**
*B. tuberculispora*	CBS 562.66 ^LT^	–	India	JN206267	JN206525
*B. tuberculispora*	CBS 570.70	–	Japan	MH859852	MH871631
*B. variabilis*	CBS 564.66 ^LT^	Excrements of human	India	JN206254	JN206528
*B. westeae*	UoMAU4 ^T^	Contaminant during attempts to culture *Laccaria* species from freshly collected sporocarps	Australia	MK959061	MK958796
*Backusella* ‘group X’	UoMAU9	–	Australia	MK959098	MK958787
*Backusella* ‘group X’	UoMAU98	–	Australia	MK959099	MK958789
*Mucor indicus*	CBS 226.29 ^ET^	–	Switzerland	HM999956	HM849690

Bold letters indicate strains and accession numbers determined in this study. CBS, Culture Collection of the Westerdijk Fungal Biodiversity Institute, The Netherlands; CNUFC: Chonnam National University Fungal Collection, Gwangju, South Korea; EML: Environmental Microbiology Laboratory (Fungarium, Chonnam National University, Gwangju, South Korea); UoMAU: National Herbarium of Victoria, Australia; URM: Micoteca Culture Collection, Universidade Federal de Pernambuco, Recife, Brazil. ^T^, ^ET, LT^, and ^NT^: ex-type, ex-epitype, ex-lectotype and ex-neotype strains, respectively.

## Data Availability

All sequences generated in this study were submitted to GenBank.
